# Applying the NIMHD research framework to assess research capacity: lessons and adaptations for use

**DOI:** 10.3389/fpubh.2026.1899861

**Published:** 2026-08-26

**Authors:** Shelby L. Langer, Aliria M. Rascón, Heather M. Ross, Julia F. Hammett, Alyssa G. Robillard, Angel B. Algarin, Felipe Gonzalez Castro, Fiorella L. Carlos Chavez, Joseph Daniels, Kelly C. Davis, Rachel Gur-Arie, Rodney P. Joseph, Sunny W. Kim, Rebecca E. Lee, Derek R. Manis, Chung Jung Mun, Eyitayo O. Owolabi, Megan E. Petrov, Shawn D. Youngstedt, Gabriel Q. Shaibi

**Affiliations:** Center for Health Promotion and Disease Prevention, Edson College of Nursing and Health Innovation, Arizona State University, Phoenix, AZ, United States

**Keywords:** health promotion, health services research, health status disparities, healthcare disparities, minority health, public health, social determinants of health

## Abstract

The National Institute on Minority Health and Health Disparities Research Framework illustrates that health and health disparities are shaped by a complex interplay of factors that operate across multiple domains and levels of influence. We sought to apply the framework in a novel way, to examine and inform the collective work of an academic research center focused on health promotion and disease prevention. Center investigators independently mapped their research onto a grid crossing five domains (biological, behavioral, physical/ built environment, sociocultural environment, healthcare system) and four levels of influence (individual, interpersonal, community, societal). We describe and reflect on two mapping exercises as a use case to strengthen practical application of the framework for research planning and evaluation. We also highlight key questions that emerged during implementation and propose potential adaptations to improve the framework’s utility for interdisciplinary teams working to address health disparities.

## 1 Introduction

The socioecological model was conceptualized to encourage a broader approach to the study of human development, beyond the individual person and intra-psychic phenomena. Its developer, Urie Bronfenbrenner, called attention to the ecological system in which the individual is embedded, defining the ecology of human development as:

*...the scientific study of the progressive, mutual accommodation, throughout the life span, between a growing human organism and the changing immediate environments in which it lives, as this process is affected by relations obtaining within and between these immediate settings, as well as the larger social contexts, both formal and informal, in which the settings are embedded* (1, p.514).

A key aspect of the model was and remains recognition that health and wellness (or conversely illness) are influenced by factors across multiple levels, from micro to macro. The model has received widespread attention and adoption given its applicability across populations and settings. It has also been adapted and expanded over the years. For example, the National Institute on Minority Health and Health Disparities (NIMHD) added another dimension or axis to the model, integrating levels of influence as per the socioecological model with *domains* of influence as per the National Institute of Aging Health Disparities Research Framework (2). The resulting model, called the NIMHD Research Framework (3) includes both levels *and* domains of influence and adds a fifth domain, the health care system. It also incorporates recognition of health outcomes across the different levels of influence and emphasizes that the multidomain drivers operate across the life course.

As described by Alvidrez et al. (3), the model was utilized in part by NIMHD to examine research on minority health and health disparities in the institute’s 2015 funding portfolio. A key finding was that much of the work focused on or addressed health outcomes at the individual level (87%) and less commonly at the interpersonal (18%), community (20%), or societal (22%) levels. Their analysis further demonstrated that the bulk of research at the individual level spanned the following domains: biological (71%), sociocultural (68%), health care system (64%), and behavioral (60%). These findings were in alignment with ongoing efforts and fostered new efforts to facilitate multi-level, multi-domain research to understand and reduce health disparities.

Beyond use of the framework by NIMHD to examine their institute’s research portfolio, the framework has been used to guide literature reviews on health disparities across a wide range of contexts. Some of these reviews have focused on disparities in specific health, disease, and healthcare contexts, for example, mental health (4), type 2 diabetes (5), postpartum weight retention (6), epilepsy care (7), liver cancer care (8), and end-of-life care (9). Others have focused on disparities affecting specific populations: sexual and gender minority older adults (10), persons living in Puerto Rico (11), and Asian and Asian American beauty service workers (12). Still others have called for expansions of the framework. Morgan et al. (13) added two intriguing levels of influence to the grid, interspecies and planetary. They cite COVID-19 as an important example of the former, classified as a zoonotic disease given that it originated in animals but then was transmitted to humans. The planetary element is also increasingly important to consider given the critical role of environmental factors in shaping and often compromising health, more so for some than others.

In short, the NIMHD Research Framework has been utilized and adapted in a variety of ways to identify and describe drivers of health disparities and to inform interventions. The present manuscript leverages the framework in a novel context, to elucidate areas of foci in the collective work of an academic research center. Founded in 2016, the Arizona State University Center for Health Promotion and Disease Prevention includes 20 transdisciplinary investigators conducting research in partnership with communities to improve health and prevent disease among vulnerable populations across the lifespan. We applied the framework to systematically characterize the center’s research portfolio. By mapping projects across levels of influence and domains of health determinants, we identified thematic areas of concentration, gaps in coverage, and opportunities to strengthen transdisciplinary collaboration. In what follows, we present our application of the framework, findings, and insights gained during the process. As such, we describe a use case along with lessons learned and suggested adaptations for adoption of this method by other investigators and organizations, to enhance the utility of the tool in guiding behavioral medicine and health disparities research.

## 2 Approach

Faculty members (*n* = 20) of the Center for Health Promotion and Disease Prevention within the Edson College of Nursing and Health Innovation at Arizona State University participated in two sequential mapping exercises during the Spring of 2025. The faculty represent a range of disciplinary backgrounds, including nursing, psychology, health communication, health policy, and kinesiology. Academic rank at the time of the activity was distributed as follows: eight Assistant Professors, seven Associate Professors, and five Professors.

### 2.1 Mapping exercise #1

Center investigators were given a blank grid paralleling the NIMHD Research Framework, crossing 6 domains and 4 levels of influence; see Table 1. Investigators were asked to enter words or phrases describing their research in relevant cells, and to leave any irrelevant cells blank. As an example, an investigator focused on health disparities among Latino children, families, and communities entered “heritability of type 2 diabetes in Latino families” in the biological-interpersonal cell. These descriptions were synthesized using content analysis. The approach was iterative. Five investigators (co-authors #2–6) coded entries, with each cell coded by two investigators. The first coding round was done independently. A second round was undertaken after exposure to the co-coder’s codes and discussion to consolidate codes as appropriate and/or resolve discrepancies. A third round was conducted by the first and last authors, to further consolidate and summarize themes. A fourth and final step was undertaken to facilitate interpretation and presentation of the codes. Two of the original coders independently analyzed the codes yielded in the previous step to identify themes corresponding to each domain, then debriefed to resolve any discrepancies and reach consensus regarding final themes for each domain. The resulting themes were defined and categorized according to the level at which they emerged.

**Table 1:** NIMHD research framework (3).

Domains of influence	Levels of influence			
	Individual	Interpersonal	Community	Societal
Biological				
Behavioral				
Physical/built environment				
Sociocultural environment				
Health care system				
Health outcomes				

### 2.2 Mapping exercise #2

Investigators were given the same grid as above but with the consolidated codes entered (Supplementary Table 1). They were asked to enter their initials in each cell that was reflected in their active research. *Active* was operationally defined as currently working in or having worked in that space during the past 3 years. Investigators were instructed not to endorse a particular cell if the work was only aspirational (e.g., if a grant proposal was under review). No constraints were placed on the work with respect to methodology or research design. Inputs were used to tabulate frequencies and create a heat map illustrating the density of representation across domains and levels of influence. Percentages per cell were calculated by dividing the number of investigators endorsing the cell by 20, the total number of investigators.

### 2.3 Subsequent step

As described in the discussion, we later added a seventh domain (row) to reflect primary *research study outcomes* as distinct from health outcomes. These entries were tabulated and added to the heatmap.

## 3 Findings

**Figure 1:**
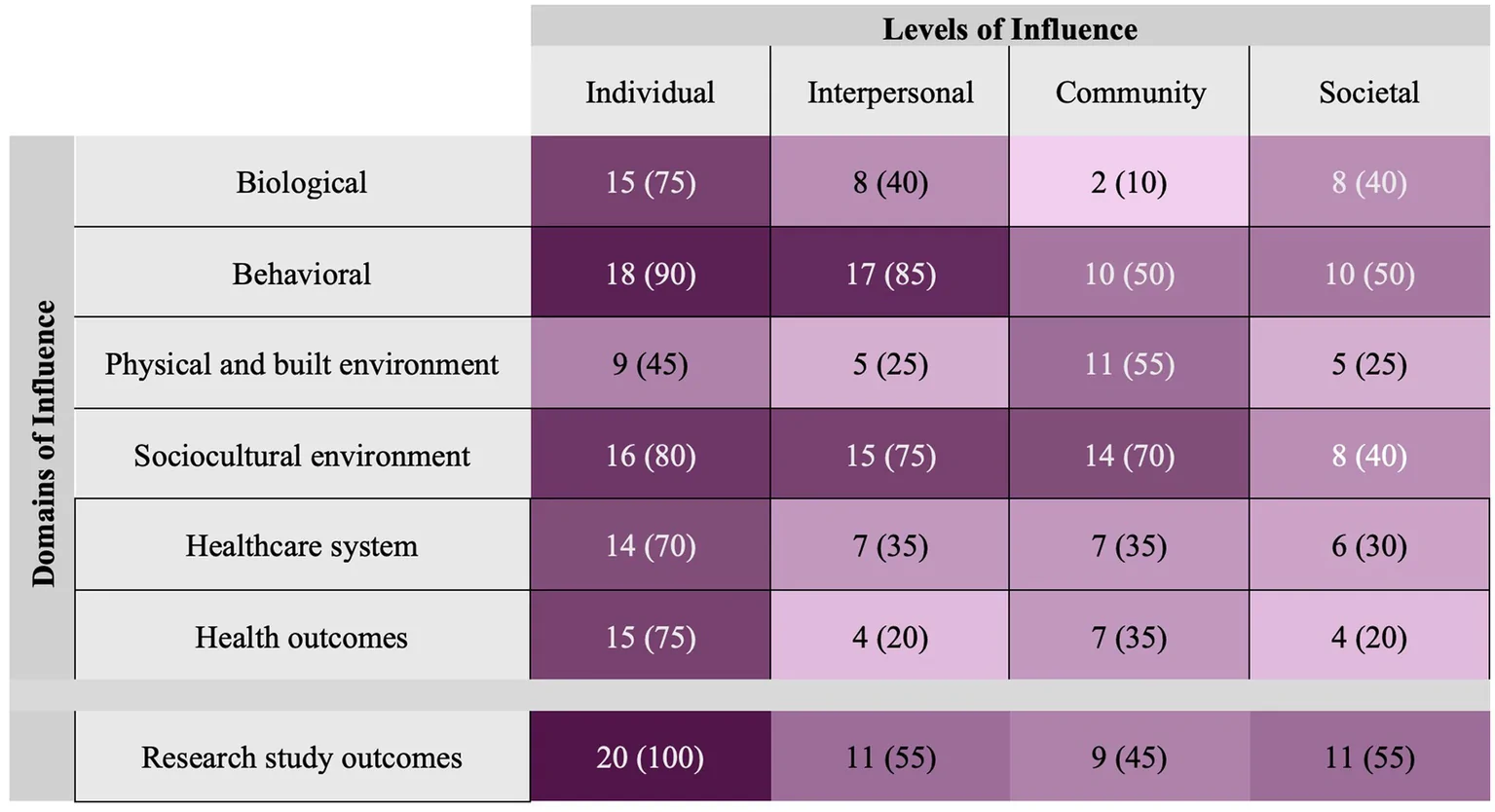
Heat map illustrating research activities across domains and levels of influence, *n*(%).

The number of cells out of 24 endorsed as representative of individual research programs ranged from three to 18 across investigators, *M* (SD) = 11.75 (4.1). Figure 1 displays the heat map illustrating frequency of activities across domains and levels of influence. As seen in the heat map, greatest density occurred at the behavioral domain within the individual (90%) and interpersonal (85%) levels of influence, followed by the sociocultural environment domain in intersection with individual (80%), interpersonal (75%), and community (70%) levels, and the biological (75%) and health care system (70%) domains at the individual level. The least-represented cells were the biological-community cell (10%) and health outcomes at interpersonal (20%) and societal (20%) levels. No cell had zero frequency.

Themes emerging from the content analysis, presented in Table 2, complemented the density mapping, as all cells (intersecting points) were represented. Themes within the biological domain were most heavily situated at the individual level, whereas those within the physical/ built environment domain were most heavily situated at the community level. Themes within the behavioral, sociocultural environment, and healthcare system domains were more evenly distributed across the different levels of influence.

**Table 2:** Identified themes within domains and levels of influence.

Domain	Theme	Levels			
		1	2	3	4
Biological	Genetics	X	X		
	Infectious disease	X	X	X	X
	Biological processes	X	X		X
	Demographics	X			
	Personality	X			
	Stress	X			
	Medications	X			
Behavioral	Health behaviors (e.g., physical activity, vaccine uptake)	X	X	X	X
	Psychosocial behaviors (e.g., coping, support)	X	X	X	X
	Violence	X	X	X	
Physical/built environment	Home/shelter	X	X	X	X
	Community spaces (e.g., bars, YMCA)			X	
	Crime and safety	X		X	X
	Work/school	X	X		X
	Access to healthy lifestyle resources (e.g., food, greenspaces)	X		X	X
	Environmental qualities (e.g., heat, air pollution)			X	
Sociocultural environment	Sociodemographics (e.g., racial identity)	X	X	X	
	Norms/customs/beliefs	X	X	X	X
	Discrimination/racism	X	X	X	X
Healthcare system	Access to services (e.g., vaccination, diabetes education)	X	X	X	X
	Communication and information	X	X	X	
	Trust and discrimination	X	X		X
Health outcomes	Healthspan and lifespan	X	X	X	X
	Disease incidence/prevalence (e.g., diabetes, obesity)	X	X	X	X
	Disease severity and symptoms (e.g., pain)	X		X	
	Disease transmission and prevention (e.g., HIV, HPV, TB)		X	X	
	Psychosocial states/conditions (substance abuse, PTSD)	X	X	X	

1 = individual, 2 = interpersonal, 3 = community, 4 = societal.

The themes highlight drivers of interest and expand on the heat map by providing information about *what* we do and in what contexts. Unlike many research centers in an academic medical setting, our center is nested in a college of nursing and is disease-agnostic, with funding from multiple NIH institutes that reflects work with diverse populations and health conditions. Notably, *infectious disease* emerged as a theme crossing all four levels of influence, highlighting collective capacity in this space. Several investigators also examine the role of *biological processes* across levels. As an example, Petrov and colleagues are conducting a longitudinal study of infant-mother pairs to examine prospective associations between infant gut microbiome development, sleep–wake patterns, and rapid weight gain (14). Themes within the behavioral domain, *health and psychosocial behaviors*, and *violence*, highlight further areas of foci. Indeed, several investigators with expertise in lifestyle behaviors are intervening to effect change not only at the individual level but also at the interpersonal, community, and societal levels, with attention to sociocultural and built environment factors. For example, Davis and colleagues are testing the efficacy of a bystander training program for staff in alcohol-serving establishments to prevent alcohol-related sexual aggression (15). Lee and colleagues are engaged in implementation testing to determine optimal delivery of an evidence-based, policy-guided physical activity and nutrition education program for preschoolers in early care and education settings (16). Joseph and colleagues are examining the role of *racism* across multiple levels of influence on physical activity and cardiometabolic risk among Black women (NCT063377080). Finally, within the healthcare services domain, there exists a strong emphasis on access to services such as vaccination and diabetes education.

## 4 Discussion

This work demonstrates a novel application of the NIMHD Research Framework as a structured tool for characterizing and evaluating the research portfolio of an academic research center. Collectively, center investigators represented every cell of the NIMHD Research Framework, with at least two faculty members working within each cell. Results of the content analysis mapping further elucidated the individual and collective research conducted by center faculty members, affording examination of our center’s efforts to understand and reduce health disparities, as well as gaps and opportunities for future work. In what follows, we comment less on these particulars (though of interest to our center) and more on the *process* of completing the evaluative exercise, as a use case for others interested in utilizing the framework in a similar manner.

Several questions arose in the process of both individual investigator entries and in the content analysis step that prompted consideration of further modifications to the NIMHD framework. To start, a key challenge in applying the framework involved the classification of constructs that did not map cleanly onto existing domains. For example, it was unclear where dispositional characteristics, such as personality traits, should be entered. The same was true for psychopathologies/ personality disorders. We ultimately put them in the biological domain / individual level cell, arguing that they represent enduring patterns. Similarly, there was some discussion about where to situate demographic characteristics. “Sociodemographics” are listed in the sociocultural domain/ individual-level cell of the NIMHD Research Framework Alvidrez et al. (3). We followed that guideline and entered gender identity, racial identity, ethnic identity, socioeconomic status, education, and income as drivers of health disparities in this same cell. We entered biological age and biological sex (i.e., sex assigned at birth) in the biological-individual cell.

Another point of discussion emerged around the entry of certain psychosocial constructs. The term “coping strategies” is listed in the behavioral-individual cell of the NIMHD Research Framework but not all coping strategies are behavioral. A psychological domain felt conspicuously absent. Indeed, this is precisely the point made by Esteban and colleagues in their reframing of the NIMHD framework, published after our coding exercise had commenced (17). They suggest expansion of the behavioral domain to include psychological factors. They note constructs such as internalized stigma and identity-related stress as examples. In the absence of this modification, we entered these types of constructs into the sociocultural environment domain, as seen in the example grid provided by Alvidrez and Barksdale (4). It is important to acknowledge that our heatmap findings are to some extent a result of these placement decisions.

A tension arose around entries for the health outcomes domain row. Some entries appeared to be more distal and, upon closer examination, not actually assessed in the specific research investigation. For example, one might collect data on HPV vaccine hesitancy but not HPV prevalence (the health outcome). This observation led to a fruitful conversation about what constitutes a health outcome and the addition of a row for *research study outcomes*, inserted above health outcomes. The addition of this row afforded examination of assessed primary research outcomes in comparison to clinically defined outcomes, often highlighting a disconnect. This disconnect relates to funding opportunities and timelines, as well as investigator timelines and pressures. Certain distal health outcomes may be highly relevant and of great interest (e.g., lifespan) but difficult to assess in primary data collection within intervention research.

Another limitation of the exercise is that it did not incorporate or summarize in any way the “how” of our collective work. We did not input information on research designs or approaches (e.g., use of technology for assessment and/or intervention delivery). These details could be incorporated and could provide insight into how, for example, technology is being used to either assess or intervene to promote health and reduce health inequities. We also lost, in the process of data reduction, specific examples of community partnerships and priority populations.

Any distillation, of course, has its advantages and disadvantages. Use of the grid afforded self-evaluation of our recent research foci, a high-level overview that will be extremely useful as we develop grant proposals and undertake other collaborative activities. We can also critically evaluate what is underdeveloped. Returning to the spirit of the socioecological model and the NIMHD Research Framework, which emphasize the need to both understand and address health disparities from multiple perspectives including the broader context in which individuals, families and communities exist, we were encouraged to find that our collective work spanned the full framework. No cell (intersecting point) was unrepresented and investigators on average reported working in 11.75 different cells. Nonetheless, with the exception of the physical/ built and sociocultural environments, the distribution of work across domains skewed toward the individual level. This was most drastic within the biological domain. It is telling that 100% of investigators reported a research study outcome, for which they might power an investigation, at the individual level. This value dropped to roughly half (45–55%) at the interpersonal, community, and societal levels. With this knowledge, we can seek out relevant expertise (which may already exist within the larger university) to help us fill gaps and to be more mindful of how current efforts could be extended to address other levels of influence within domains. The goal is not necessarily full coverage of all cells within the framework, but instead coverage that aligns with our strengths, strategic priorities, and capacity for making an impact.

We offer a replicable tool for use by other investigators and organizations in a similar capacity, along with lessons learned and suggestions for modification. If time is constrained and a quick and dirty overview is desired, the density mapping activity is faster and less taxing though providing coarser information as compared to the content analysis. In both processes, however, we encountered questions of fit and placement, to some extent due to lack of specificity in the framework but also a product of our different disciplinary backgrounds and the fact that certain constructs may not fit neatly into a specified cell. Indeed, as seen in Table 2, many themes were recurrent.

To date, the framework has by and large been used retrospectively, e.g., to examine extant literature bases. Here, we used it to examine our collective current work to inform and guide future efforts such as collaborations, research ideas, and grant proposals. A unique contribution is the addition of a research study outcomes row which allows for examination of these constructs and assessments in comparison to health outcomes. A disconnect between the two may point to gaps in empirical evidence linking proximal and more distal outcomes. Funding mechanisms and timelines may constrain what can be both rigorously and feasibly assessed within a single research study or even research career. Within the context of a transdisciplinary center focused on promoting health and preventing disease, it can be argued that the absence of disease is the most salient health outcome. In the field of prevention science, this approach is often framed around reducing risk factors and enhancing protective factors. A disconnect between research outcomes and health outcomes may warrant adaptations to the framework that consider health outcomes defined more broadly as well-being or human flourishing rather than simply the absence of disease.

The adapted framework could be used in a variety of other ways, from scaffolding individual student learning plans and courses to developing training grant proposals and other larger proposals such as program projects and center grants. Finally, it could be used as a lens with which to disseminate study findings and to communicate the breadth of an academic center or other organization’s work.

## Data Availability

The original contributions presented in the study are included in the article/supplementary material, further inquiries can be directed to the corresponding author/s.
