# Supplementary material for: Applying the NIMHD research framework to assess research capacity: lessons and adaptations for use
Source: Front Public Health. 2026 Aug 26;14:1899861. doi: 10.3389/fpubh.2026.1899861 (PMC13553885; doi:10.3389/fpubh.2026.1899861)
Supplement: Supplementary file 1 [file Table_1.docx]

Supplemental Table 1. Consolidated grid

|  | | Levels of influence | | | |
| --- | --- | --- | --- | --- | --- |
|  |  | Individual | Interpersonal | Community | Societal |
| Domains of influence | Biological | Demographics   - sex - age   Personality traits  Genetics and genomics  Biological and physio processes  Disease status  Psychopathology/ personality disorders  Stress  Medications | Infectious disease transmission  Dyadic and family (dis)concordance in biological processes  Heritability  Physiological impact of caregiving | Infectious disease/ community transmission and exposure  Herd immunity | Environmental exposures Population vaccination uptake  Rates of disease |
|  | Behavioral | Health behaviors  Psychosocial behaviors | Couple/ family/ network functioning  Social support  Communication  Coping  Shared health behaviors  Sex and violence  Interpersonal and protective health behaviors (HPV spread, condom use)  Joint problem-solving and shared decision-making | Vaccine uptake  Engagement in disease prevention and health promotion practices  Violence  Discrimination | Policies and laws (e.g., stealthing laws, immigration, regulations governing care delivery)  Norms (e.g., drinking)  Executive orders  Human rights  Guidelines/ recommendations/ public health initiatives  Trade agreements for migrant work |
|  | Physical/ built environment | Home environment  Access to basic needs   - shelter - safety - transportation | Living/ household/ work/ school environment | Public/ social settings (bars)  Social network environment  Homeless shelters and informal settlements  Walkability/ active transport  Crime and safety  Access to healthy lifestyle resources (food, greenspace)  Faith-based organizations  Health-focused, community-based organizations (YMCA, one-in-ten)  Other community-based organizations and clubs (barbershops)  Community gardens  Neighborhood deprivation  Air pollution  Heat  Levels of illumination | Historical and structural inequities (e.g., redlining, Apartheid)  Policy and urban planning  Safety  Access to public transportation  Resource security/ stability (water, electricity)  Housing  Employment opportunities  Access to healthy lifestyle resources (food, greenspace) |
|  | Sociocultural environment | Sociodemographics   - gender identity - racial identity - ethnic identity - SES/ education/ income   Sexual orientation  Beliefs  Language | Social/ family norms, beliefs, customs and preferences  Racism/ discrimination  Workplace climate  Family income | Community norms/ beliefs/ customs/ attitudes (stigma)  Resilience  Connectedness/ cohesion/ isolation  Racism/ discrimination  SES  Language | Norms/ traditions/ practices/ beliefs (e.g., discrimination and racism) |
|  | Health care system | Access to healthcare  Insurance status  Treatment preferences  Trust in health system  Health literacy | Patient-provider communication/ interactions  Shared decision-making  Cultural competence and humility  Racism/ discrimination  Screening practices | Healthcare facilities (hospitals, clinics) and related supportive care services  Specialized clinics/ services for marginalized conditions or populations (Ryan White)  Vaccination information/ services  Digital health libraries | Policies and laws  Politics  Access to health care and coverage  Quality of care  Regulations and regulatory bodies  Institutionalized racism  Availability of treatment and preventive biologics/ medications/ approaches |
| Health outcomes | | Life/ healthspan  Diseases/ conditions   - ADRD/ cog impairment - Cancer - HIV - TB - HPV - Diabetes - NAFLD - Stroke - Obesity - Pain - Substance use disorder - Cardiometabolic disease   Psychosocial outcomes   - Depression - Anxiety/ PTSD/ distress - QOL/ well-being - Stigma - Coping - Resilience | HIV suppression/ transmission  TB transmission  Intergenerational obesity and diabetes  Homicide  Post-partum depression | Disease prevalence/ incidence  Lifespan and healthspan  Transmission rates (viral load, TB)  Vaccination rates  Disease-related morbidity and mortality  Substance use disorder/ overdose  Victim justice outcomes | Disease incidence/ prevalence  Health disparities  Premature morbidity and mortality  Economic burdens |
|  | | | | | |
| ***Research study outcomes (raw investigator entries, not consolidated)*** | | | | | |
| Research study outcomes | | Glucose tolerance  Quality of life  Relationship satisfaction  Psychological distress  Physical activity  Abdominal adiposity  Vaccine hesitancy  Alcohol/ substance use  HbA1c level  Sleep health  Weight gain  Diabetes Prevention Program (DPP) attendance  Sexual aggression  Sexual health indicators  HIV viral load  Treatment completion  HIV testing  Physical pain  Heart rate variability parameters  Depressive symptoms  TX adherence biomarkers  Housing status  Cardiometabolic disease risk  Social isolation  Cognitive decline  PrEP uptake  Resilience | Family glucose tolerance  Family engagement  Family coping  Relationship stability  Intimate partner violence  Social network # discussion partners, # collaborators, # advice partners and density  Health system engagement  Family sleep health  DPP reach, diffusion, impact within family system  Family and peer support  Shared cultural values  Public safety engagement  Social network stigma | Access to diabetes prevention services  Access to HIV, TB and OST services  Costs and cost effectiveness payer perspective  Access to vaccines  Access to genomic medicine  Trust in healthcare  Access to and engagement in diabetes self-management education and support programs  Bar-level policies  Healthcare literacy  Improving rural health  Community driven stigma  Employment/ work  Public safety response to mental health crisis | State-level diabetes rates  Costs to cost effectiveness ratio societal perspective  Vaccination uptake  Genomic medicine implementation  Reduced T2D-related complication rates  State-level DPP retention rates  Police reports across AZ  Future of U.S. agriculture  Migrant labor health and welfare regulations  Rates of homelessness  Comparative population-level outcomes  Health service utilization  Stroke incidence |
